# Queer German Superstitions

**Published:** 1885-10

**Authors:** 


### Queer German Superstitions.

LUCK IN FISH SCALES—TELLING FORTUNES WITH MELTED LEAD—THE BIBLE.

A true German would never think of going to bed on Sylvester Abend before the ushering in of the new year. Were he to be guilty of such forgetfulness a year of sickness and ill-luck would surely attend him. From the emperor down to the most humble of his subjects, the night is spent in reading the psalm-book, recounting old family stories, dancing, in singing, conviviality and eating. So, too, does every loyal German buy a carp and feast upon it with brown sauce and potatoes at this time. The fishmongers frequently make enough money on Dec. 31st to support them a year. For, since every one must have a fish, and since thousands are sold, advantage is taken of the custom to send the price up to eight and ten marks per fish. The scales of the fish must be saved, and punctually at midnight secured in a sealed packet ; a small number are put in the pocket-book, in which there must be some money. This is sure to bring the possessor much wealth during the ensuing year. The fish is cooked in some sweet sauce, and is quite delicious. The roe brings with it excellent good luck.

At 11 o'clock each member of the family melts some lead in a shovel over a hot stove, and when it is thoroughly liquefied it is poured quickly into a bowl of water. Naturally, it assumes all manner of queer formes and shapes, which a lively imagination can weave into various types. Early on New Year's morning the forturne-teller calls, and with these leaden effigies spread out before her, proceeds to read the fortunes for the coming year. Now, it is a singular inter-reaction of the psychic with the physical that these forms should often partake largely of the substance of that which occupied the mind when the lead was poured into the water. For just at this time one must make a wish. Whether the insensible metal does really take on the forms, or whether the imagination, governed by the wish, causes them to assume such forms the reader must judge for himself

At midnight each one jumps into a chair, turns about, jumps down and shouts and then jumps up again. Each member of the family embraces, and the streets are filled with "Prosit Neu Jahr" from the mouths of the passers-by. Then the Bible is again brought out, and without looking, the finger is placed on a certain verse, which becomes the text for the next twelve months. The young women congregate together in one room, and, opening the stove door, expect to find the faces of their future husbands in the sparkling flame. Others, with a similar object in view, peer into the looking-glass just as the clock begins to strike twelve. For ghosts the Germans have all possible respect, and their legendary tales are full of quaint, weird, fireside stories.—*Philadelphia Press.*

